# Melanoma Mimicking Malignant Peripheral Nerve Sheath Tumor with Spread to the Cerebellopontine Angle: Utility of Next-Generation Sequencing in Diagnosis

**DOI:** 10.1155/2018/9410465

**Published:** 2018-06-28

**Authors:** Katie Fox Hanson, Paul Birinyi, Ronald Walker, Constantine Raptis, Rebecca Chernock, Jeroen Coppens, Katherine E. Schwetye

**Affiliations:** ^1^Department of Pathology, Saint Louis University School of Medicine, 1402 S. Grand Blvd., St. Louis, MO 63104, USA; ^2^University of Tennessee Health Science Center, Department of Neurosurgery/Semmes-Murphey Clinic, USA; ^3^Department of Otolaryngology, Saint Louis University School of Medicine, 1402 S. Grand Blvd., St. Louis, MO 63104, USA; ^4^Mallinckrodt Institute of Radiology, Washington University School of Medicine, 660 S. Euclid Ave., St. Louis, MO 63110, USA; ^5^Department of Pathology and Immunology, Washington University School of Medicine, 660 S. Euclid Ave., St. Louis, MO 63110, USA; ^6^Department of Neurosurgery, Saint Louis University School of Medicine, 1402 S. Grand Blvd., St. Louis, MO 63104, USA

## Abstract

Cutaneous spindle cell malignancy is associated with a broad differential diagnosis, particularly in the absence of a known primary melanocytic lesion. We present an unusually challenging patient who presented with clinical symptoms involving cranial nerves VII and VIII and a parotid-region mass, which was S100-positive while lacking in melanocytic pigment and markers. Over a year after resection of the parotid mass, both a cutaneous primary lentigo maligna melanoma and a metastatic CP angle melanoma were diagnosed in the same patient, prompting reconsideration of the diagnosis in the original parotid-region mass. Next-generation sequencing of a panel of cancer-associated genes demonstrated 19 identical, clinically significant mutations as well as a high tumor mutation burden in both the parotid-region and CP angle tumors, indicating a metastatic relationship between the two and a melanocytic identity of the parotid-region tumor.

## 1. Introduction

Melanoma is heterogeneous in both clinical behavior and histomorphological appearance and may resemble other types of tumors, particularly in the case of a spindle cell malignancy [1–4]. Desmoplastic melanoma often lacks melanin pigment and is positive for S100 by immunohistochemistry but negative for other markers of melanocytic differentiation.

Malignant peripheral nerve sheath tumor (MPNST) is a spindle cell neoplasm which may appear morphologically similar to spindle cell and desmoplastic melanoma. Like these subtypes of melanoma, MPNST may show patchy S100 positivity in the absence of melanocytic markers. The differential diagnosis of an S100-positive spindle cell lesion in a cutaneous location is challenging given these overlapping features [1, 2, 4].

Lentigo maligna melanoma (LMM) arises in the sun-damaged skin of older adults; early clinical identification of LMM on the face can be difficult due to the presence of multiple solar lentigines [5].

We present a case for which next-generation sequencing of a panel of cancer-associated genes established a relationship between an intracranial melanoma and a prior right parotid region tumor. Further, the assay identified FDA-approved therapies targeted for specific genetic alterations in this patient. We suggest that genomic analysis may serve both diagnostic and predictive functions in similar, challenging cases.

## 2. Case Presentation

**Figure 1:**
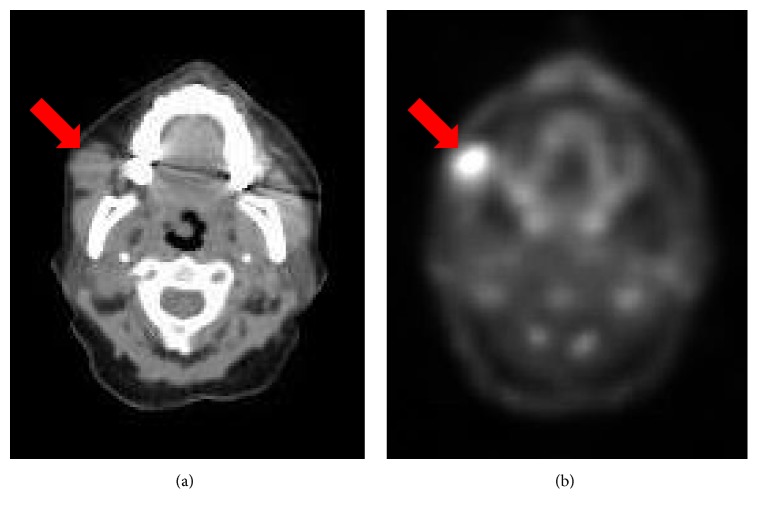
CT (a) and PET (b) images of the FDG-avid mass, centered along the anterior aspect of the right masseter muscle, not involving parotid.

**Figure 2:**
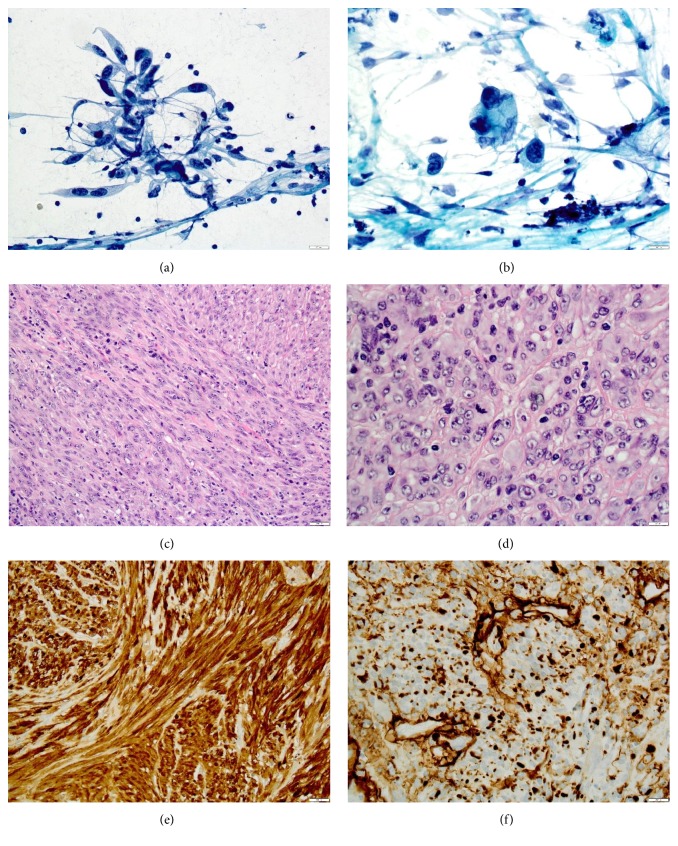
(a), (b) Pap-stained cytologic preparations of the right parotid-region mass; (c), (d) hematoxylin and eosin-stained sections of the parotid-region mass; immunostains for S100 (e); collagen IV (f).

A 63-year-old female sought medical intervention for a painless, firm, mobile mass within her right cheek. For the previous year-and-a-half, she had experienced right facial nerve paralysis, which progressed to facial numbness and progressive hearing loss. A PET-CT scan showed an FDG-avid 2.2 x 2.0 cm mass centered along the anterolateral aspect of the right masseter muscle without parotid gland involvement (Figure 1). An MRI of the lesion indicated enhancement of the right trigeminal nerve from its origin to the point where it entered Meckel's cave along with enhancement of the right facial nerve from the internal auditory canal to the middle ear. A fine needle aspiration of the mass showed clusters of atypical spindled cells with elongated, irregular nuclei; the tumor was diagnosed as a malignancy consistent with neural or mesenchymal origin (Figures 2(a) and 2(b)). A total right parotidectomy with selective resection of the facial and trigeminal (mandibular division) nerves was performed. Histopathologic review showed a tumor adjacent to, but not primarily involving, the parotid gland, characterized by a proliferation of spindle cells, many with multiple nuclei, grouped in interwoven fascicles and heavily interwoven with lymphocytes (Figure 2(c)). Nuclei were prominent and markedly pleomorphic, and the mitotic index was high (28/10 high-power fields; Figure 2(d)). Immunohistochemical stains showed S100 to be strongly and diffusely positive (Figure 2(e)); collagen IV was 2+ positive around individual tumor cells (Figure 2(f)); Mart1/MelanA and HMB-45 were negative (not shown). Pancytokeratin, CK5/6, p63, desmin, CD34, and the mutant protein BRAF V600E also were negative. The tumor was diagnosed as a poorly differentiated MPNST. There was no evidence of metastatic tumor in the additionally submitted lymph nodes. The patient subsequently completed radiation therapy.

**Figure 3:**
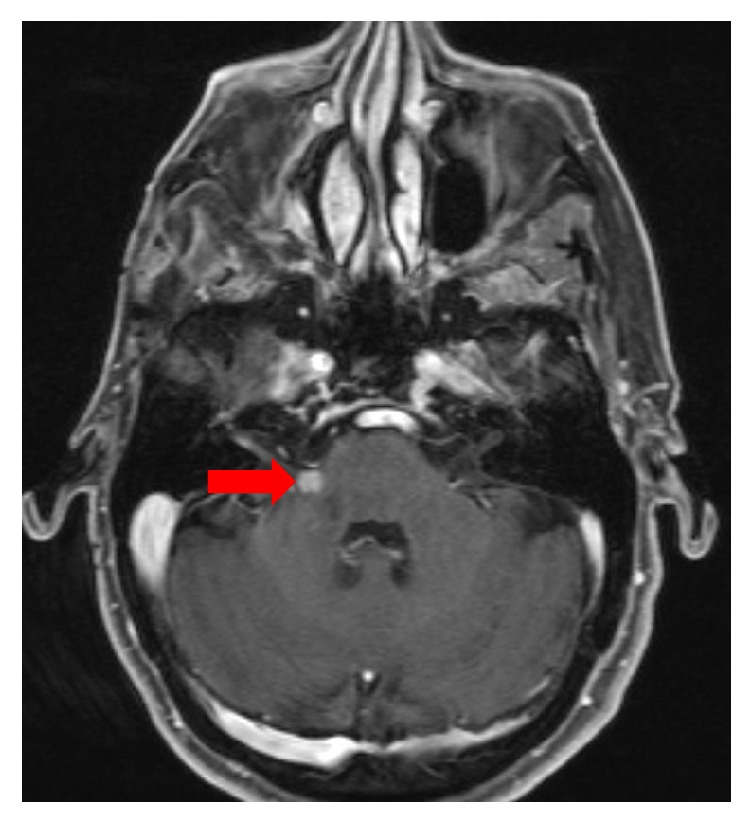
T1-weighted image with contrast, highlighting new right-sided cerebellopontine angle lesion (arrow).

**Figure 4:**
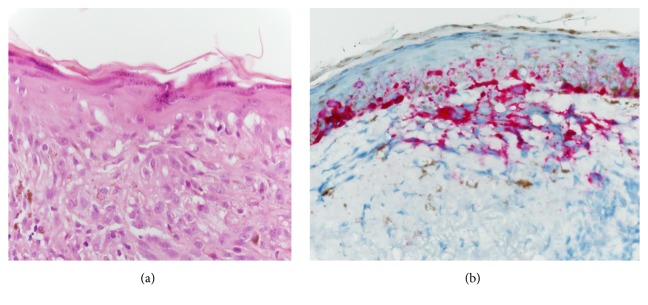
Hematoxylin and eosin-stained section (a) and MART1/Melan A immunostain (b) demonstrating lentigo maligna melanoma of the right cheek (400X power).

Approximately eight months after the initial resection, the patient presented with severe hearing loss in her right ear and difficulty with walking and balance. An MRI revealed a 6.4 mm, contrast-enhancing lesion at the right cerebellopontine (CP) angle (Figure 3) and a similar 3.8 mm lesion slightly distal running along the 7^th^ cranial nerve. At this time, a 1.5 x 1.5 cm irregular pigmented lesion also was noted on her right cheek. A punch biopsy of this lesion revealed LMM (Figure 4). In this biopsy, the Breslow depth was 0.25 mm; neither ulceration nor dermal mitotic activity was noted. Additionally, although neither lymphovascular nor perineural invasion was identified, the possibility was raised that the original parotid-region mass represented a metastatic melanoma instead of a separate primary MPNST.

**Figure 5:**
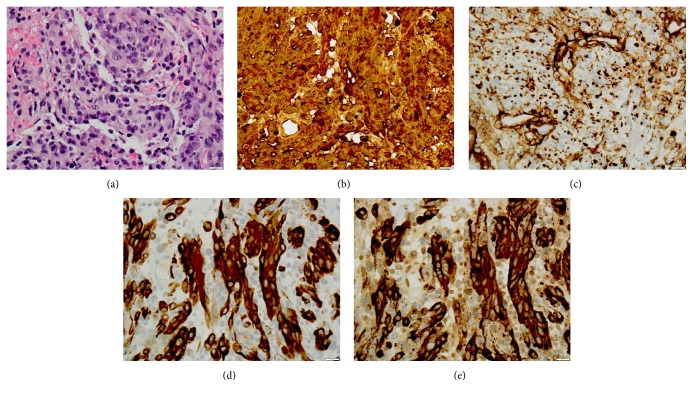
(a) Hematoxylin and eosin-stained section of CP angle tumor; immunostains for S100 (b), collagen IV (c), Mart1/Melan A (d), and HMB-45 (e).

The patient then underwent resection of the CP angle tumor as well as cranial nerve 7 approximately 23 months after the parotid-region mass was resected. Histopathologic review demonstrated spindled-to-epithelioid cells with pleomorphic nuclei (Figure 5(a)). In several areas, fine, golden-brown pigment was observed. The mitotic index was 4/10 high-power fields, and a Ki-67 immunostain showed an 18% index of proliferation. Similar to the original parotid-region mass, the tumor showed strong and diffuse positivity for S100 (Figure 5(b)), and there was weak focal reactivity for collagen IV (Figure 5(c)). Unlike the originally resected parotid-region mass, the CP angle lesion exhibited strong and diffuse positivity for Mart1/MelanA and HMB-45 (Figures 5(d) and 5(e)) and was diagnosed as malignant melanoma.

She also underwent a wide excision of her right cheek melanoma, which revealed residual melanoma with a Breslow depth of 0.55 mm. Again, no dermal mitotic activity, ulceration, lymphovascular, or perineural invasion were noted. The tumor was again positive for Mart1/MelanA, as was demonstrated in the original biopsy.

Given the uncertain relationship between the original parotid-region and the CP angle masses, next-generation sequencing of a panel of 300+ genes was performed on both the parotid-region mass and the CP angle lesion (Foundation Medicine; Boston, MA). Both tumors showed high tumor mutation burden (TMB), and each showed over twenty potentially clinically significant genomic alterations; among these, they shared nineteen (Table 1).

## 3. Discussion

This case highlights several important clinical and diagnostic challenges in a complex patient. Our patient presented with facial paralysis and numbness, prompting imaging studies which revealed a spindle cell mass in the parotid-region with morphological and immunohistochemical features consistent with an MPNST for which she underwent resection as well as radiation therapy. At that time, the patient had no known melanocytic lesions. Months later, a pigmented lesion on her right cheek was clinically recognized, biopsied, and diagnosed as LMM. Imaging also identified a small (<1 cm) CP angle lesion that was resected and diagnosed as melanoma.

One obvious possibility was that the right cheek LMM represented the primary tumor, and the right parotid lesion as well as the CP angle tumor represented metastatic sites. Although lentigo maligna melanoma is sometimes considered more “benign” on the spectrum of melanoma subtypes, it is often associated with lymphovascular spread and metastasis. One recent study examined the risk factors associated with subclinical spread of invasive melanoma [6]; multivariate analysis identified tumor localization on the head and neck and history of previous treatment, both of which are relevant features in this patient, as well as age > 65 years and mitotic activity > 1/mm^2^. Tumor histologic type and Breslow depth, interestingly, were not risk factors for subclinical spread of invasive melanoma.

Since the right parotid lesion demonstrated a unique morphologic appearance and immunohistochemical profile (negative for both Mart1/MelanA and HMB-45), the question as to how these three lesions were possibly related prompted additional diagnostic studies, namely, next-generation sequencing using a broad, cancer-associated panel. Of the three tumor sites, the parotid-region and the CP angle tumors contained adequate cellularity required to perform the assay and were both shown to not only demonstrate the high tumor mutation burden characteristic of melanomas on chronic sun-exposed skin [7] but also share at least nineteen clinically significant alterations. Importantly, these alterations include a truncating mutation in* NF1* (R2517^*∗*^). These data strongly suggest that the parotid-region and CP angle tumors are genetically related given the vanishingly small probability that nineteen identical alterations arose by chance.

This patient's tumors thus might be classified as belonging to the “*NF1*” subtype in the proposed genomic classification of cutaneous melanoma from the Cancer Genome Atlas project [8]. As compared with other subtypes,* NF1* subtype melanomas show higher tumor mutation burdens and the strongest UV mutation signature [9]. Mutations in* NF1* are more common in melanomas occurring on chronically sun-exposed skin or in older patients [10, 11], melanomas with higher mutation burden [11] or wild-type for BRAF and NRAS [10, 12, 13], and the clinicopathologic subtype, desmoplastic melanoma [14–16]. Interestingly, a mechanistic link has been proposed between NF1 loss and an MPNST-like melanoma phenotype [17].

Three of the nineteen shared genomic alterations, including high TMB; NF1 R2517^*∗*^; and MTOR E1799K, are associated with FDA-approved therapies and with investigative therapies in clinical trials. Although to date the patient has required no further oncologic treatment, a priori knowledge of specific genomic alterations in these tumors provides options should additional therapy be indicated.

Another interesting aspect of this case is the unusual presentation of malignant melanoma in the CP angle. The MRI showing spread of melanoma along the 7^th^ and 8^th^ cranial nerves suggests that the melanoma reached the CP angle via the leptomeninges. Though malignant melanoma has a high propensity to metastasize to the brain, it is usually by hematogenous spread [2]. Additionally, malignant melanoma to the CP angle is rare, accounting for only 0.2-0.7% of neoplasms in this location [18]. In fact, Gerganov et al. report that, at the time of their writing, there were only seventeen cases of intracranial metastasis of malignant melanoma to the CP angle reported in literature [19].

In conclusion, this patient had an unusual presentation of malignant melanoma. The use of next-generation sequencing was valuable to clarify the relationship between the parotid-region mass and the CP angle lesion. This case highlights the potential use of next-generation sequencing in the diagnosis of traditionally difficult-to-assess tumors.

## Figures and Tables

**Table 1:** Genomic alterations in the parotid-region and CP angle tumors.

Parotid-region tumor	CP angle tumor
*NF1 R2517* ^*∗*^	*NF1 R2517* ^*∗*^
*MTOR E1799K*	*MTOR E1799K*
*CDKN2A p16INK4a P81L*	*CDKN2A p16INK4a P81L*
*EPHA3 D832N*	*EPHA3 D832N*
*ERBB4 E563K*	*ERBB4 E563K*
*FANCA R1084C*	FANCA Q869^*∗*^, *R1084C*
*FLT1 E72K*	*FLT1 E72K*
*GNAS P201L*	*GNAS P201L*
*IKZF1 splice site 851-1G>A*	*IKZF1 splice site 851-1G>A*
*LRP1B H2277Y, S1992F, splice site 13325-1G>A*	*LRP1B H2277Y,* Q763^*∗*^*, S1992F, splice site 13325-1G>A*
*MAGI2 E1166K*	*MAGI2 E1166K*
*NOTCH2 Q2325* ^*∗*^	*NOTCH2 Q2325* ^*∗*^
*PIK3CG D192N*	*PIK3CG D192N*
*SNCAIP E661K*	*SNCAIP E661K*
*SPTA1 R1281C, R1659* ^*∗*^	*SPTA1* Q1720^*∗*^*, R1281C, R1659*^*∗*^
*TP53 S241F*	*TP53 S241F*
*Tumor mutation burden TMB-High; 186 muts/Mb*	*Tumor mutation burden TMB-High; 225 muts/Mb*
**FAM123B C635** ^**∗**^	ATM R3047 ^*∗*^
**EGFR amplification**	SLIT2 R617 ^*∗*^
**TERT promoter -124C>T**	

Italic: identical mutations in parotid/CP angle.

Bold: unique to parotid.

Underline: unique to CP angle.
